# Larvicidal Activity and Histopathological Effect of *Averrhoa bilimbi* Fruit Extract on *Aedes aegypti* from Surabaya, Indonesia

**DOI:** 10.1155/2020/8866373

**Published:** 2020-08-01

**Authors:** Etik A. Rohmah, Sri Subekti, Marcellino Rudyanto

**Affiliations:** ^1^Institute of Tropical Disease, Universitas Airlangga, Surabaya 60115, Indonesia; ^2^Master Program of Tropical Medicine, Faculty of Medicine, Universitas Airlangga, Surabaya 60132, Indonesia; ^3^Faculty of Fisheries and Marine, Universitas Airlangga, Surabaya 60115, Indonesia; ^4^Faculty of Pharmacy, Universitas Airlangga, Surabaya 60115, Indonesia

## Abstract

*Averrhoa bilimbi* has been long thought to have biological activity. The aim of this study was to determine the activity of primary and secondary metabolites from *A. bilimbi* fruit extract on *Aedes aegypti* larvae mortality and midgut histopathology. Experiment was performed to third-instar *Ae. aegypti* larvae collected from Surabaya, which then exposed to *A. bilimbi* crude fruit extract at various concentration for 24 hours. After exposure, larvae were evaluated of its mortality and fixed in 2.5% neutral buffer formalin before processed and sectioned into histological slides and stained with hematoxylin-eosin (HE). Statistical analysis was performed using *Spearman rank* correlation to determine histopathological damage on midgut of *Ae. aegypti* larvae. Phytochemical screening of *A. bilimbi* crude fruit extract found that it contained saponins, tannins, and terpenoids. Minimum concentration able to induce mortality on *Ae. aegypti* larvae (LC_50_) was 977 ppm, while LC_90_ was at 1380 ppm. Severe alteration of larvae midgut was found after 24 hours exposure to 2000 ppm extract. Features of damage mostly found in larvae midgut were disruption of the microvilli, columnar cell vacuolization, epithelial nucleus crossed midgut lumen, and basal membrane damage. Damage caused by fruit extract in midgut of *Ae. aegypti* third instar larvae inhibited development of larvae. This study reported first finding of histopathological effect of *A. bilimbi* fruits extract on *Ae. aegypti* larvae midgut. Result of study was expected to contribute to better understand extract bioactivity of this plant to be applied as natural larvicide for *Ae. aegypti*.

## 1. Introduction

Dengue hemorrhagic fever (DHF) has long caused burden to public health of Indonesia. This disease is mostly found in tropical and subtropical regions of the world. In Indonesia, DHF is an endemic disease found to infect people all-year long but peaked mainly during rainy season, which is the optimum condition for mosquito reproduction [1].

Historically in Indonesia, DHF emerged firstly in Surabaya and Jakarta at 1968 with total case number of 58 and case fatality rate (CFR) at 41.3%. After its first emergence, DHF spread to other big cities. Surabaya region was ranked first on the number of DHF cases in East Java Province of Indonesia [2].

On the last three consecutive years, the number of DHF cases in Surabaya has gradually lowered, but still persisted; total of 938 cases was found in 2016, 325 cases in 2017, and 321 cases in 2018 [2]. This was possibly due to high level of migration or mobilization of residents from DHF nonendemic area to DHF-endemic area, or travelling from rural to urban area (posttravelling fever) [3]. *Aedes aegypti* is main vector of DHF epidemic in Surabaya. In addition, transsexual transmission among mosquitoes after copulation and transovarian transmission from parents to offspring also spread dengue virus further.

Control of dengue virus transmission can be conducted by various methods; however, the main method is control of vector population, which is mainly performed by applying inhibitor chemicals. However, repetitive chemical control can cause new issue, such as leaving residue that can contaminate the environment. Thus, alternative methods to control *Ae. aegypti* population, which is more ecofriendly, can be applied [4]. One of those methods is by using natural chemical compounds extracted from plants with potential as effective and ecofriendly control of mosquito larvae. This results in the exploration of natural larvicide from plants. Various plants have been identified to contain bioactive compounds with insecticidal activity such as saponins and terpenoids.

Larvicidal compounds can enter insect body through body wall and respiratory and digestive organs. Body wall can absorb high level of toxic materials. Toxic materials are relatively easier to be absorbed due to hydrophobic and lipophilic properties of insect cuticle, causing nonpolar bioactive compounds to be readily passed through the cuticle and to enter the insect body [5].

Previous studies had tested various plants as *Ae. aegypti* larvicidal agents; however, they are commonly purposed to determine activation and effective concentration to induce high level of mortality of larvae. Pharmacological activities of *Averrhoa bilimbi* have been studied from its fruits, leaves, and flowers. It was found to be an antidiabetic, antihyperlipidemic, antimicrobial, hepatoprotective, anthelmintic, and antioxidant agent [5]. Furthermore, it was also found to be useful in malaria [6]. But study on their physiological larvicidal effect is still limited. The aim of this study was to determine the effect of *A. bilimbi* crude fruit extract on mortality and midgut histopathological alterations of *Ae. Aegypti* larvae. Larvicidal compounds contained in *A. bilimbi* extract was expected to be able to be used as an alternative method to control DHF vector, which potentially is more ecofriendly, effective, and low-cost with wide availability in nature.

## 2. Materials and Methods

### 2.1. Plant Extraction

Fruits of *Averrhoa bilimbi* were obtained from a personal garden, planted organically without added insecticide. Fruit was cut into small pieces and airdried without direct sun. Dried fruit was crushed into powder using blender. Extraction was performed using maceration by immersing powdered fruit in 96% ethanol for one day. Resulting filtrate was concentrated using rotary evaporator at boiling point of 40-50°C, 70 rpm speed, and 0.7 bar pressure, until volume was reduced to a third of the initial volume. Concentrated extract was heated in oven at temperature 40°C until the condensed extract containing bioactive compounds was obtained.

### 2.2. Larvae Rearing

Eggs of *Ae. aegypti* were collected using ovitrap method from Sawahan district, Surabaya, East Java province, Indonesia. This survey was approved by Bakesbangpol of Surabaya city (no. 070/8029/436.8.5/2019). Larvae were then brought to Entomology Laboratory, Institute of Tropical Disease, Universitas Airlangga, for rearing to be hatched as first-generation larvae. This generation was maintained under optimum condition (room humidity 65-80%, water temperature 28-30°C) until it hatched and developed to be third-instar larvae.

### 2.3. Larvicide Test

Larvicide test in the current study was conducted based on standard method [7]. Larvae used were third-instar larvae of *Ae. aegypti* collected from the field. Extract was made into several concentrations: 500, 1000, 1500, and 2000 mg/L using tap water as solvent. Negative control used tap water. For each treatment, 100 ml of extract was placed into a plastic container. Then, 25 third-instar *Ae.aegypti* larvae were placed into each container and observed for 24 hours. Larva mortality was evaluated at the end of the 24-h exposure.

### 2.4. Histopathological Evaluation

After exposure, the larvae were fixed in 5 ml 2.5% formaldehyde for 24 hours in room temperature. Larvae sample was then dehydrated using a series of graded alcohol and embedded in paraffin. The larvae were sectioned using microtome at 4 *μ*m thickness to obtain midgut section. Sections were stained using hematoxylin-eosin (HE) and evaluated under light microscope.

### 2.5. Statistical Analysis

Larva mortality was analyzed using *post hoc* LSD test to determine the difference of each group and probit analysis to determine LC_50_ and LC_90_. Histopathological alteration of larvae midgut was analyzed using qualitative and *Spearman* rank correlation test.

## 3. Results and Discussion

### 3.1. Larvicide Test

Mortality of third-instar *Ae. aegypti* larvae from Surabaya was found to differ significantly between groups exposed to different concentrations of *A. bilimbi* extract (*p* < 0.01). Larva mortality after exposure to various concentrations of *A. bilimbi* extract is presented in Table 1.

Extract concentrations that induced mortality of third-instar *Ae. aegypti* larvae were started from 1000 mg/L, resulted in 57% mortality; 1500 mg/L resulted in 94% mortality; to 2000 mg/L resulted in 100% mortality, after exposure for 24 hours. Observation under the microscope of third-instar *Ae. aegypti* larvae exposed to *A. bilimbi* extract at 2000 mg/L found that it showed these reactions towards extract; larvae body elongated (±7 mm, from initial length of ±5 mm), body numbed, and abdominal lateral spikes detached from cuticle. Larvae exposed to negative control was found to be morphologically undamaged; entirety of body was intact and larvae size was unchanged (±5 mm). On the other hand, larvae exposed to positive control showed a different reaction compared to both negative control and extract treatment; larvae body tended to shrunk from its initial length (±4 mm from initial length of ±5 mm), cuticle was damaged, and abdominal lateral spikes were detached. Dead larvae were found to be turned to white color, be rigid, and body length tended to be longer than its initial length.

Based on larvae mortality after exposure, probit analysis showed that lethal concentration 50 (LC_50_) of *A. bilimbi* extract was at 1061.275 mg/L (range 927.294-1170.705 mg/L), while LC_90_ was 1461.255 mg/L (range 1308.955-1794.687 mg/L) (Table 2). The addition of *A. bilimbi* extract at concentration 1308.955-1794.687 mg/L possibly resulted in 90% mortality of *Ae. aegypti* larvae collected from Surabaya, East Java Province, Indonesia.

### 3.2. Histopathology Test

**Figure 1:**
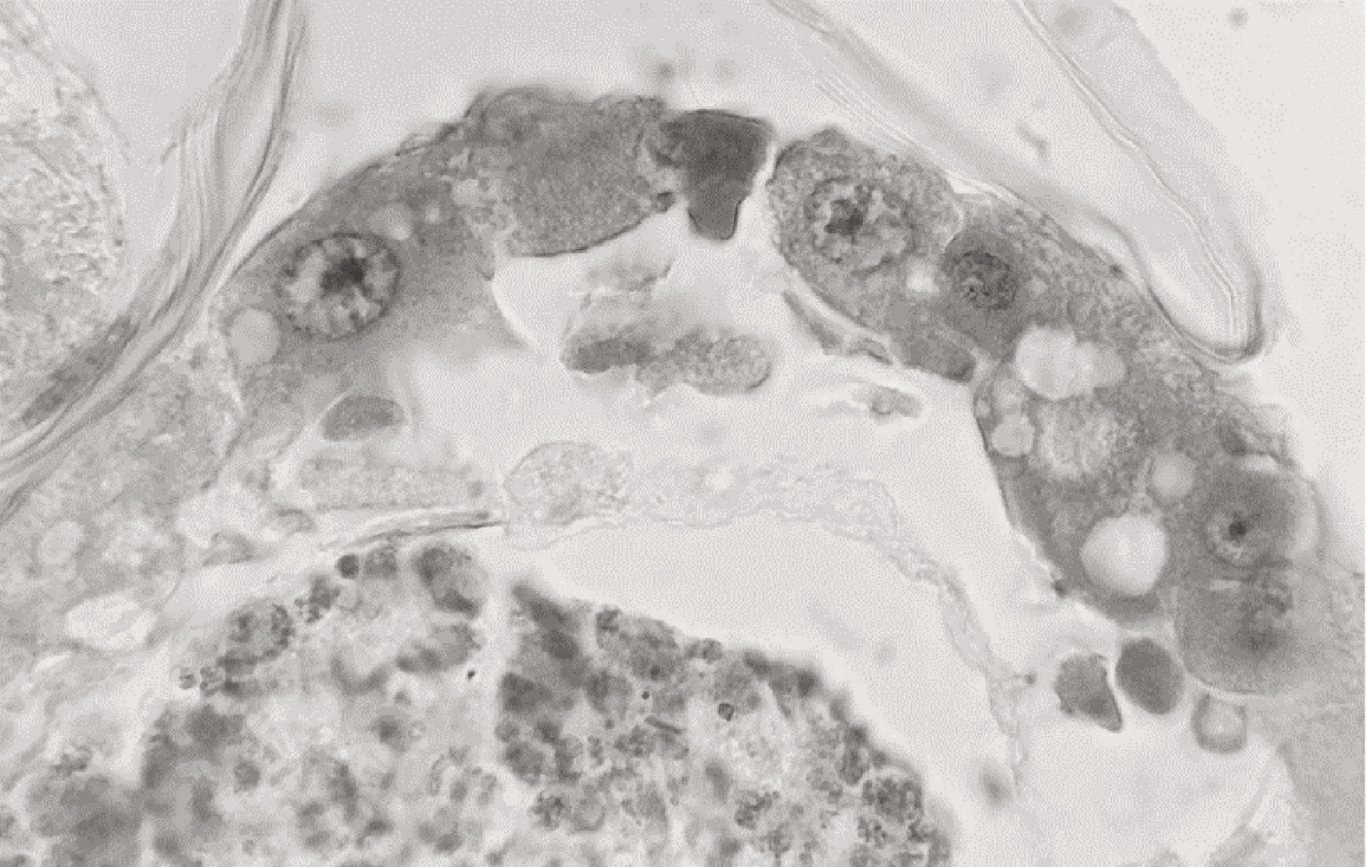
Cross-section part of the midgut of 3th instars larvae of *Ae. aegypti* larvae midgut exposed to 2000 ppm *Averrhoa bilimbi* extract for 24 hours. lm: lumen, v: vesicle, n: nucleus, mv: microvilli, mb: basal membrane.

Larvae exposed to *A. bilimbi* fruit extract was examined histologically. Damage was found mainly in epithelial cells of the midgut (Figure 1), such as cytoplasmic vacuolization, protruding cytoplasm, disruption of the microvilli, columnar cell vacuolization, and basal membrane damage.

Figure 1 shows the changes that occur in the midgut cells which became irregular and damage to the microvilli at the top of the cell. The cytoplasm experiences protrusion, which is indicated by an arrow.

**Figure 2:**
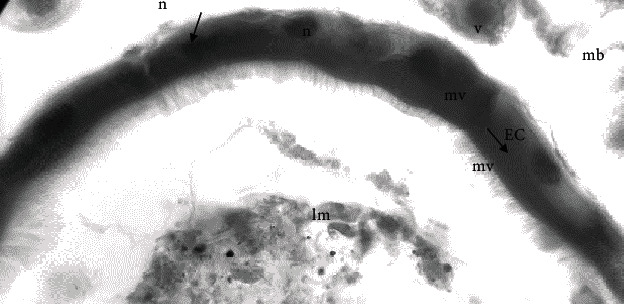
Cross-section histopathology of the midgut of the control group showing epithelial cells (EC) with microvilli (mv).

Compared to the control group (Figure 2), the midgut epithelial cells did not repair damage.

## 4. Discussion

Based on this manuscript discussion, fruits extracts from A. *bilimbi* can kill the larvae with lethal mortality rates, 1061.275 ppm (50% of deaths from the total larvae) and 1461.255 ppm (90% of the total larvae that died) in Table 2. This means that it has the effectiveness of killing larvae from Surabaya Indonesia. The larvae showed damage in the larval midgut epithelial as well as histopathological damage which are presented in Figures 1 and 2. Further research needs to be done to see its effects on other mosquito larvae genera such as Culex, Anopheles, and Mansonia which act as mosquito-borne diseases.

Histopathology evaluation of third-instar *Ae. aegypti* larvae exposed to *A. bilimbi* fruit at various concentrations showed that the midgut was affected by extract. Various damages were found in the midgut, especially in the epithelium cells, microvilli, and basal membrane. These damages were due to larvicidal compounds contained in *A. bilimbi* fruit extract, which were saponins [8] that explained that *Averrhoa bilimbi* fruits contained saponins. Saponins have potential as larvicide and work as stomach poison in *Ae. aegypti* larvae by lowering surface tension of the mucosa membrane in the digestive tract, making it easier to be damaged. Damage occurred in the midgut of larvae mainly because of various functions occuring in this place, such as digestion, nutrition absorption, ion transport, and osmoregulation [9]. The midgut is mainly composed of basal membrane-supported epithelium cells that layer body wall. Similar to other insects, larvae stomach functions not only in digestion but also in chemical and mechanical defense against pathogens [10].

In the negative control, larvae could still perform normal activities, such as eating, active moving, and floating in water surface. As they did not get interference or stress from extract, larvae were still able to live normally. Given no extract, no diffusion occurred in larvae body, resulting in zero mortality, but due to *A. bilimbi* fruit extract components solely. This research is in accordance with [11], who explained that the results showed that the strong pigmentation of larval midgut was still observed even in the presence of PTU (a strong phenoloxidase inhibitor), indicating that the midgut darkening was not related to melanization or resulted from other mechanisms, such as tissue injuries caused by the extract, or due to the accumulation of leaf extract into the larval midgut, and one of them experienced disruption of the gut homeostasis due to the partial detachment of the peritrophic matrix and extensive tissue disorganization in the midgut.

And this is in accordance with [12] who explained that acceleration of diffusion occurred based on difference of concentrations of dissolved materials during the process. The higher the difference of concentration, then the faster diffusion will occur, resulting in higher stress to the larvae. No difference of concentration means that no diffusion occurred, such as that in in larvae of negative control. Histopathological evaluation in the negative control (0 ppm) did not show damage to all tissues in the midgut (Figure 2). Midgut 0 ppm *Ae. aegypti* larvae has a single layered columnar epithelium with a midgut cell nucleus in the middle and microvilli is well developed.

At 2000 ppm, extract caused severe damage to the larvae midgut. Epithelium cells and basal membrane were found to be damaged. Most nucleus was observed left out of broken epithelium cells towards the lumen (Figure 1). Damaged vesicle was observed to be protruded to the lumen and the cytoplasm formed bubble-like structure. The damage of the digestive tract due to larvicidal is mostly found in the midgut part, because food digestion and nutrition absorption occur in this part [13], and as explained that the damage to digestive cells in the midgut of *Ae. aegypti* larvae caused by the *S. terebinthifolius* leaf extract may have impaired the digestive and absorption processes in the larval midgut, compromising survival, and disrupting larval mosquito development. The regenerative cells play an important role in the development since they start their division in the last larval instar and finish in the early pupal stage, resulting in renewing of intestinal epithelium, an essential step in metamorphosis [11].

This was caused by toxic effect of primary metabolites in extract towards columnar epithelium layering midgut. This caused disruption of enzyme secretion and food digestion, subsequently making larvae to have energy deficit, which could lead them to die [14]. This was caused by toxic effect of primary metabolites in extract towards columnar epithelium layering the midgut. This caused disruption of the enzyme secretion and food digestion, subsequently making larvae to have energy deficit, which could lead them to die [14]. Previous studies found that other parts of *A. bilimbi* could potentially act as natural larvicide. Extract of *A. bilimbi* flower could induce changes in the midgut of *Anopheles barbirostris* larvae, causing damage on peritrophic membrane, epithelium cells, and basal membrane, and saponins enter the larvae body through the digestive tract; thus, it can function as stomach toxin that disrupts nutrition absorption. Other compounds, terpenoids, are antifeedant that averse larvae to feed, consequently inducing mortality [8]. Extract of *A. bilimbi* leaf and fruit could damage the villi and cause malformation of the epithelium cells in the midgut of *Ae. aegypti* larvae [15]. Histological evaluation of the midgut evaluation showed effects of secondary metabolites in *A. bilimbi* fruit, possibly saponins of terpenoid groups, which resulted in damage based on its psychological roles and physicochemical characteristics.

Important limitation of this study should be noted: *A. bilimbi* extract used was crude extract, not result of purification or isolation, thus concentration of *A. bilimbi* fruit extract was high. Further study can be conducted to evaluate the damage to secretory cells in the midgut using periodic acid Schiff-stained histological sections. In addition, the effect of extract acidity on the ionization of *Ae. aegypti* midgut larvae should be further studied. Lastly, the effect of extract on other species of vector mosquito species (*Culex*, *Anopheles*, and *Mansonia*) should also be explored, as local specific affects test results for both larvae and extract applied.

## 5. Conclusion

Based on mortality and histopathological evaluation after 24-hour exposure, extract of *Averrhoa bilimbi* fruit could be used as larvicide to *Aedes aegypti*. The crude extract of fruit has LC_50_ of 0.977 g/L on third-instar *Ae. aegypti* larvae, while at concentration 1.38 g/L, extract had caused permanent damage to larvae midgut. Pathological effect found in the midgut was damage in the basal membrane, columnar cells, and cytoplasm.

## Figures and Tables

**Table 1:** Mortality of third-instar *Ae. aegypti* larvae from Surabaya after being exposed to various concentrations of *Averrhoa bilimbi* extract for 24 hours.

Conc. of *A. bilimbi* extract (ppm)	Number of *A. aegypti* larvae per application (*N*)	Percentage of larvae mortality (%)
Control	25	0
500	25	0
1000	25	57
1500	25	94
2000	25	100

**Table 2:** LC_50_ and LC_90_ of *Averrhoa bilimbi* extract based on mortality of third-instar *Ae. aegypti* larvae after 24 hours of exposure.

Strain	LC_50_ (ppm)	Lower bound (ppm)	Upper bound (ppm)	LC_90_ (ppm)	Lower bound (ppm)	Upper bound (ppm)
Surabaya	1061.275	927.294	1170.705	1461.255	1308.955	1794.687

## Data Availability

The data used to support the findings of this study are available from the corresponding author upon request.
